# Supplementary material for: Electrically driven reprogrammable phase-change metasurface reaching 80% efficiency
Source: Nat Commun. 2022 Mar 30;13:1696. doi: 10.1038/s41467-022-29374-6 (PMC8967895; doi:10.1038/s41467-022-29374-6)
Supplement: Supplementary file 1 — Supplementary information [file 41467_2022_29374_MOESM1_ESM.pdf]

Supplementary Information for

Electrically driven reprogrammable phase-change metasurface reaching 80% efficiency

Sajjad Abdollahramezani,† Omid Hemmatyar,† Mohammad Taghinejad,† Hossein Taghinejad,† Alex Krasnok,‡ Ali A. Eftekhar,† Christian Teichrib,¶ Sanchit Deshmukh,§ Mostafa A. El-Sayed,‖ Eric Pop,§,⊥,# Matthias Wuttig,¶ Andrea Alù,‡,@ Wenshan Cai,†,△ and Ali Adibi*,†

†*School of Electrical and Computer Engineering, Georgia Institute of Technology, Atlanta, Georgia 30332, United States*

‡*Photonics Initiative, Advanced Science Research Center, City University of New York, New York, NY 10031, United States*

¶*Physikalisches Institut IA, RWTH Aachen, 52074 Aachen, Germany*

§*Department of Electrical Engineering, Stanford University, Stanford, California 94305, United States*

‖*Laser Dynamics Laboratory, School of Chemistry and Biochemistry, Georgia Institute of Technology, Atlanta, Georgia, 30332, United States*

⊥*Department of Materials Science and Engineering, Stanford University, Stanford, California 94305, United States*

#*Precourt Institute for Energy, Stanford University, Stanford, California 94305, United States*

@*Physics Program, Graduate Center, City University of New York, New York, NY 10016, United States*

△*School of Materials Science and Engineering, Georgia Institute of Technology, Atlanta, Georgia 30332, United States*

E-mail: ali.adibi@ece.gatech.edu

Note 1: 3D electrothermal simulation model of metadevices

We perform the electrothermal analysis for the meta-switch by creating a 300 μm $\times$ 300 μm $\times$ 1000 μm 3D finite element model in COMSOL Multiphysics (see Fig. S2a). To consider a real practical model, we assume the heterostructure device is attached to a 500 μm silicon substrate. In our simulations, Electric Currents module is employed to calculate the voltage and current distribution in the device, and Heat Transfer in the Solid module is used to predict the temperature profile. The two modules are coupled via Joule heating and the temperature dependence of material properties.

Electrical phenomena in the device are modeled via Poisson and continuity equations, $\nabla.[\sigma(x, y, z, t)\nabla V] = 0$, in which σ is the electrical conductivity of the microheater material. If the thermal characteristic of the microheater is modeled as a first-order system comprising a parallel thermal resistance (R_t) and thermal capacitance (C_t), the cooling rate follows an exponential relation with a time constant of $\tau = R_t C_t$. R_t and C_t depend on the length and the width of the microheater as well as the thermal properties of the ambient. Therefore, judicious selection of the contributing materials and design of the heterostructure metadevice can guarantee successful repeatable, reversible, and multi-state phase transformation of the PCM. W is used as a well-known refractory material for the heating element due to the moderate electric conductivity, good thermal conductivity, high temperature endurance, good metal barrier properties, and high resistance to electromigration[1,2]. The electrical resistivity obtained from four-point probe measurements (at room temperature) of a 50-nm-thick sputtered W is 500 nΩ.m, which is consistent with experimental data reported in the literature[1]. We set 0.0015 1/K as the resistivity temperature coefficient of W as the temperature and resistance of the microheater increase when the voltage across the microheater evolves with time[3,4]. The governing transient heat transfer equation is described by[5]

$$C_s \rho \frac{\partial T}{\partial t} + \nabla.(-k\nabla.T) = Q_s \tag{1}$$

where C_s is the specific heat capacity, ρ is the density, k is the thermal conductivity, T is the time- and space-dependent temperature, and Q_s is the Joule heat source per volume. For all external boundaries, we set open thermal boundary conditions except for the bottom of Si substrate and top surfaces where a heat flux condition with ambient temperature of $T = 20$ °C and the heat transfer coefficient of 5 W/m^2K is considered. We assume the convective cooling by ambient and radiation loss are insignificant. To model the heat fluxes and temperature gradients at the interfaces, thermal boundary resistance (TBR) is applied at interior boundaries. The measured TBRs in units of m^2k/GW are as follows[6–14]: Au/Al_2O_3: 48, Al_2O_3/GST: 28, HfO_2/Si: 10. For other interior boundaries TBR is assumed 25 m^2k/GW, which is typical for many systems[15,16]. The thermal conductivity of GST depends both on the temperature and phase as detailed in the literature[17].

Quantitative analysis of the crystallization kinetics of the GST element exposed to the annealing pulses is crucial for precise control of the properties of the light. Fig. S2b represents the crystallization fraction of GST in different intermediate states as a function of temperature at the center of the GST film. Each data point that correlates with an experimental voltage pulse in Fig. 3b is calculated as follows: first, reflectance spectra for 21 different intermediate states of GST are obtained from separate full-wave electromagnetic simulations, then, the more resembled one to the measured reflectance spectra is found, finally, a FEM simulation is carried out to find the induced temperature due to the stimulating voltage pulse with characteristics detailed in Methods. We use an effective medium theory to calculate the optical constant of intermediate states of GST (see Note 3 for details).

Temperature uniformity is a crucial measure for successful operation of large-scale phase-change metasurfaces. The magnified spatial distribution of the reflected beam from the meta-switch in Fig. 2a in two different states of GST, i.e., C-GST and A-GST, at $\lambda = 1440$ nm are shown in Fig. S2c. These rather uniform profiles across the device are expected since

a nonpatterned GST patch with an even topography is used, which significantly reduces the surface-induced scattering loss. This evenness is also suggested by the electrothermal simulations presented in Fig. 1b showing a fairly uniform heat profile is across the microheater at the end of the set/reset pulse. In contrast to the A-GST case, where a trace of jags appears at the circumference after amorphization, the C-GST case has a more uniform boundary. This follows the rationale that long nature of the set pulse allows uniform formation and growth of crystalline zones in the background of A-GST; however, the fast reset pulse randomly leaves some partially amorphized zones at the edge of the metasurface far from the center of the microheater.

To further study the heat distribution evenness, the temperature map of the microheater (studied in the main text) in the cross section and across the center of the GST film perpendicular to the current flow are shown in Figs. S2d and S2e, respectively. Evidently, the minimum temperature of GST upon stimulation with a set/reset pulse is more than 250 °C/770 °C confirming that GST can indeed be fully crystallized and melted, respectively. In addition, the difference between the maximum and minimum induced temperature across the center of the GST film is less than 10 °C and 20 °C for the former and latter cases, respectively, which suggests complete phase transition of GST after the process. Since large probing pads act as heat sink during Joule heating process, heat dissipates faster along the microheater. One way to gain higher temperature uniformity is increasing the edge clearance between the metasurface and the pads by elongating the microheater. Figures S2f and S2g display the temperature profiles for a device with 1.6 μm clearance between the edge of the metasurface and that of the probing pads. This modification can address the undesired effect of temperature deviation and decay rate from the center of microheater.

In addition, we explore the correlation between the metadevice size and the spatial nonuniformity in the reflection value through eletrothermal simulations conducted for a metasurface with a larger aperture size that can tolerate 20% reflectance contrast at $\lambda =$ 1640 nm. According to Fig. 3a(ii), such a contrast corresponds to 40% crystallization change

between the center (with 100% crystallization fraction) and edges (with 60% crystallization fraction) of the GST film. In order to comply with this criterion, the temperature variation should be limited to ~ 50 °C between the center and edges of the GST film, with temperatures equal to ~ 260 °C and ~ 212 °C, respectively (see Fig. S2b). Though microheaters with larger footprints improve the temperature uniformity across the device, for the sake of miniaturization, we have considered the area of the microheater 15% larger than the metasurface area (similar to what we experimentally demonstrated). For a fair comparison, the multiphysics simulation is carried out under the application of a 200 μs-long (or equivalently at the speed of ~ 5 kHz) double exponential waveform that necessitates a peak voltage of 6.7 V. Figure S3 displays the temperature distribution in a cross section in the center of the GST film at the end of the set pulse. The isothermal contours reveal a ~ 50 °C temperature variation across an 80 μm $\times$ 80 μm metasurface upon applying an electrical pulse with the predefined properties.

Addressing individual meta-atoms is an important yet challenging step that can empower dynamic multifunctional metasurfaces to prevail over conventional spatial light modulators (based on liquid crystal or microelectromechanical structures). This would be more demanding when dealing with subwavelength meta-atoms whose reconfiguration mechanism depends on Joule heating. Since stimulation of each meta-atom relies on the precise control over biasing of the laterally extended pixelated heater elements, the performance measure of the metasurface is affected by the lossy nature of the wires network. At the expense of losing addressability over one direction, this negative effect could be fairly alleviated by relying on a 1D array of resistive nanoribbons (i.e., nanoheaters) homogeneously heating meta-atoms in the orthogonal direction. In this way, gate biassing can be applied to the end faces of individual micro-electrodes through compact contact pads without interfering with the optical beam. Along this direction, a simple prototype of tunable gated field-effect metasurfaces consisting of 96 independently addressable elements with pitch size of 400 nm has been recently demonstrated[18]. This along with the potential of robust sub-micrometer

width tungsten electrodes as a high-temperature high-speed heater hold the promise for the realization of dense micro-electrodes externally controlled through an electrical signal (e.g., a voltage provided by a printed circuit board).

To figure out the feasibility of local addressing of GST cells using Joule heating, electrothermal simulations are carried out for a 1D array of heterostructure ribbons (600-nm-width, 10-μm-long) comprising tungsten nanoheaters and the reflective meta-atoms (see Fig. S4). We study the impact of the reset pulse on the phase transition of GST by applying a 200-ns-long 3.1 V electrical pulses to the micro-electrode. Figure S4a shows the heat profile in a cross section at the center of the array at the end of the reset pulse. It is evident that the center pixel can be homogenously heated above 630 °C that ensures complete re-amorphization. The transient temperature profiles in Fig. S4b shows that during the entire heating process, the temperature of neighboring GST cells remains well below the onset crystallization temperature (i.e., $\sim$ 160 °C). Such a negligible thermal crosstalk between the neighboring elements guarantees the successful addressability of the phase-change metasurface at the pixel level.

Note 2: Effective medium theory for the optical characterization of intermediate states of GST

Amongst the existing effective-medium theories, we use the Lorentz-Lorenz relation to model the optical constants of GST in the intermediate states as follows[19]:

$$\frac{\epsilon_{eff}(\lambda)-1}{\epsilon_{eff}(\lambda)+2} = m \times \frac{\epsilon_C(\lambda)-1}{\epsilon_C(\lambda)+2} + (1-m) \times \frac{\epsilon_A(\lambda)-1}{\epsilon_A(\lambda)+2}, \quad (2)$$

in which $\epsilon_A(\lambda)$ and $\epsilon_C(\lambda)$ are the permittivities of A-GST and C-GST, respectively, at wavelength λ, and m, ranging from 0 (associated with 0% crystallinity (or A-GST)) to 1 (associated with 100% crystallinity (or C-GST)), is the crystallization fraction of GST. The

optical constant of A-GST and C-GST (annealed A-GST at 180 °C for 15 minutes in a rapid thermal annealing (RTA) chamber under vacuum condition) are obtained using Tauc-Lorentz and Cody-Lorentz models[20] in ellipsometric measurements. The optical constant of GST for 6 different crystallization fractions are represented in Fig. S5. The refractive index of loss-less ALD-deposited Al_2O_3 obtained from ellipsometric measurements in the near-IR spectral range is ~ 1.74.

Note 3: Cyclability performance of phase-change metasurfaces

Endurance limit of PCMs is a critical aspect of phase-change metasurfaces that influences the reliable operation of reconfigurable optical devices over a multitude of switching cycles. Though several degradation/failure mechanisms of PCMs have been reported in the literature, elemental segregation and void formation are the most appreciated ones[21,22]. The former stems from the electromigration of the constituent elements that negatively affects the crystallization kinetics and properties. The latter is attributed to the change in the mass density of PCMs upon transition in the structural phase, which finally leads to the formation cracks due to the mechanical stress. Besides these effects, the deviation observed in the reflectance response of C-GST after initial switching cycles is likely due to the formation of bigger nucleated islands with different fractions of two known crystalline phases of GST, i.e., face-centered-cubic and hexagonal[23], and inter-diffusion of layers during repeated cycles of melt-quenching as a result of material fatigue. Similar initial cycle-to-cycle variation has been also reported in phase-change memories[22,24]. To ensure high reproducibility with a stabilized switching operation, an initial conditioning treatment is imperative, in which a single reset pulse followed by a train of power decreasing pulses are iteratively employed for a few times[25,26]. Inspired by the mature technology of phase-change memories, the following strategies can also be considered to improve the switching cycle lifetime: 1) Using an array

of downscaled phase-change cells (instead of a continuous patch) each of which experiencing successful amorphization across its volume upon applying the reset pulse. Complete melting process at every reset switching and remixing of the elements fairly decrease the spatial segregation[21]; 2) Leveraging interfacial phase-change structures where a superlattice configuration comprising of stacked Sb_2Te_3 and GeTe layers enables solid–solid (rather than typical solid-liquid-solid) phase transformation between the crystalline and amorphous states[27]; 3) Using high-quality growth processes (such as molecular beam epitaxy and atomic layer deposition) to improve the interface qualities and reducing the grain sizes that facilitate uniform conversion across the material volume[28,29]; 4) Exploiting thermal layers such as graphene to reduce atomic migration during the phase transformation[30]; 5) Discovering phase-change alloys showing zero-mass density change upon phase transition (such as GaSb and GeTe-CuTe)[31]; 6) Minimizing the exposure of the PCM cell to the ambient by tight encapsulation of the PCM cell in a diffusion-free medium with high thermal conductivity. Since switching cyclability is also closely linked to the device design and the compounds surrounding the PCM, appropriate selection of materials and clever design of heterostructures can improve the number of switching cycles in phase-change metasurfaces. Despite the infancy of electrically reconfigurable phase-change nanophotonics, so far, a few thousand cycles of switching have been demonstrated in optical phase-change memories after which the device performance starts to degrade[32].

Note 4: Extending the tunable range of GST-based metasurfaces

In principle, the effective dielectric constant of PCMs at different crystallization levels is modelled with an effective medium theory such as Lorentz-Lorenz relation. Continuous tuning of GST from amorphous to fully crystalline, through an infinite number of intermediate states characterized by unique optical constants, is offered by this model. However, in prac-

tice, such a luxury is found to be quasi-continuous, i.e., a limited number of intermediate states is accessible, depending on the dynamics of phase-change and properties of surrounding compounds. The crystallization mechanism of GST is defined as nucleation dominated[31]; upon applying an external stimulus, nuclei are randomly formed in the amorphous matrix followed by their omnidirectional growth due to the temperature rise until they impinge each other and form a uniform crystalline island in the bulk of the PCM. In addition to the stochastic nature of nuclei formation[33], phase transformation is also influenced by the intrinsic properties of the substrate and capping layer materials as well as the roughness of embedding media[31]. While in our current work, 4 distinguishable, reliable, and repeatable intermediate states are demonstrated through a single-pulse programming, up to 34 levels of crystallization levels has been recently reported in integrated photonic phase-change memories using a dual-pulse programming technique[34]. To increase the intermediate levels, adoption of the following strategies for fine control over the fraction of crystalline nuclei is imperative. First and foremost is employing of an iterative signal programming scheme in which: i) a sequence of gradually decreasing power set pulses, or ii) a packet of consecutive pulses with the same power is followed by sequential verification steps, that is the reflectance measurement of the metasurface[35,36]. Each of these pulses initiates a partial crystallization in a feedback loop leading to precise control over the crystallographic state of GST. In addition to the iterative programming, leveraging a pulse shaping technique with controllable amplitude width of the electrical pulse(s) can be beneficial for the multi-level operation of the phase-change cell[34]. Along with such coding and signal processing strategies, other plans such as i) leveraging an array of miniaturized cells formed by engineered heterostructures that comprise ultrathin layers of the PCM, ii) synthetizing new PCMs characterized by minimized change in configurational entropy between amorphous and crystalline states, and iii) investigation of compositionally tuned PCMs with engineered nucleation and growth rates that relax the stochastic behavior of GST can be pursued in future efforts.

Note 5: Active tuning of the phase response

In addition to the global control over the reflection amplitude and phase supported by the phase-change meta-switch and meta-deflector, respectively, the studied hybrid metasurface holds the promise for local tuning of the individual cells for beam steering and beam forming applications. Figure S10 depicts active control over the phase property of the reflected light where upon continuous transition of the state of GST from amorphous to full crystalline, more than 180° phase shift with small reflectance variation is achieved. With the promising simulation results on addressing individual pixels of a phase-change metasurface using Joule heating process (see Fig. S4), such optical performances well compete with those provided by the state-of-the-art technologies such as gate tunable transparent conductive oxides[37].

Note 6: Data visualization based on manifold learning

Thanks to the fascinating cognitive capabilities of humans through visual perception, data visualization techniques for representation of datasets governed by complex relations have been considered as a useful tool. t-SNE, as the state-of-the-art non-parametric, nonlinear dimensionality reduction technique with the major purpose of data visualization[38–40] has attracted significant interest in this area. Despite its usefulness in visualizing high-dimensional data, in our study, unveiling the nature of the governing modes through the inspection of t-SNE plots is not straightforward, if not impossible, though the electromagnetic modes that governs the spectral responses of the metasurface (or equivalently shape the local and global structure of datapoints in the high-dimensional space) is important hidden information that is likely preserved during the transformation. In other words, no direct link exists between the results of the spatially extended clusters in the latent space and the physics of the problem. The essence of using t-SNE is to trustfully represent complex datasets with intrinsically high dimensions in low-dimensional spaces while preserving as relevant information as possible[41,42]. However, t-SNE can uncover hidden structures in the high-dimensional

dataset through capturing much of the local structures, while also revealing global structures such as the presence of clusters[38,43]. This is possible mainly due to two unique features of t-SNE. First, this algorithm seeks to preserve pairwise distances between data points by translating Euclidean distances between data points in the high-dimensional space into conditional probabilities that represent similarities[39]. In projection of the high-dimensional data to the low-dimensional space, t-SNE tends to position the points on a plane (or hyperplane) such that the pairwise distances minimize a cost function, which is a measure of the similarity between two probability distributions[42]. In addition, t-SNE fairly ameliorate the crowding problem that is distinguished by the overlap of projected datapoints in the center of the low-dimensional map due to the excessive attractive forces between moderately distant datapoints in the original space[38]. In this regard, distinctly isolated clusters of similar datapoints sharing multiple features can be readily identified in the low-dimensional space, though t-SNE is not a dedicated clustering algorithm[44–46]. In our case, the algorithm forms two widely separated natural clusters of similar points, i.e., for A-GST and C-GST cases (blue and red dots in Fig. 5a). The minimum overlap between two clusters implies that despite the variation of the structural parameters, each state of GST can only provide a specific cluster of datapoints, which are different in nature from those in the other cluster. Noteworthy, the axes of a t-SNE plot are abstract scores describing complex curved paths in the original space and are not meant to be straightforwardly interpretable in terms of the axis/units of the original high-dimensional space. This is the reason why t-SNE plots without scaling are also common in the literature. In addition, unlike simple linear dimensionality reduction algorithms such as principal component analysis (PCA) whose plot axes are weighted linear combinations of the original dimensions, as specified by the principal eigenvectors of the covariance matrix, the axes in t-SNE plots do not convey any specific information[42].

To expand our discussion, we investigate the evolution of electric field distribution for three simulated metasurfaces with different structural parameters and with A-GST and C-

GST (see Fig. S11a). For each state of GST, comparable mode profiles can be observed for all 3 randomly selected samples. While the top row illustrates the excitation of SR-SPP mode at the interface of the Au nanodisk and Al_2O_3 layer, the field enhancement in the bottom row mainly occurs at the tips of the Au nanodisk and interface of the Au back-reflector due to the excitation of LR-SPP. The reduced-dimensional responses are indicated in the 3D latent space in Fig. S11b using color-coded shapes. While this should not be interpreted as the physics extraction capability, at least it gives a tangible insight on the linking between some randomly selected datapoints within one cluster to the mode profile of the corresponding meta-atoms.

We would like to conclude that while visual representation of high-dimensional data in a low-dimensional space is imperative for exploratory data analysis, certain attention should be paid to prevent some common misreading of t-SNE plots. 1) The relative distance between well-separated clusters in the low-dimensional space does not provide any intuition. In fact, t-SNE does not retain distances but probabilities, so Euclidean distances in high-dimensional and low-dimensional spaces do not provide any useful measure about the response of the metasurface. 2) The relative sizes of the clusters in t-SNE plots do not convey any specific meaning. This stems from the fact that t-SNE tends to expand/contracts the dense/sparse data clusters of the high-dimensional space in the projected embeddings. 3) The topology of generated clusters in the latent space does not provide any information about the nature of the dataset in the original dimension. The algorithm hyperparameters fairly affect the elongation, curvature, or clumping of the generated clusters. 4) Successive runs of the t-SNE algorithm do not necessarily generate similar outputs. This follows from the rationale that some hyperparameters like prepexility, a measure of the effective nearest neighbors, play a key role in the optimization process. Despite its multiple strengths in data visualization, t-SNE has some weaknesses. The main functionality of t-SNE is for visualization purposes so using it as a pre-processing machine-learning approach is tricky, if not improper. Also, t-SNE has a low performance on datasets with high intrinsic dimensions especially given the

nonconvexity of the cost function that requires optimization of several hyperparameters.

Note 7: Assessing the feasible physical responses using machine learning

To study the range and degree of feasible responses offered by A-GST and C-GST metasurfaces, we leverage a hybrid approach consisting of both dimensionality reduction and i) convex hull or ii) one-class support vector machine (SVM) techniques. We limit our discussion to the main outcomes of these approaches, as the working principles and theoretical frameworks are detailed in our previous work[47]. In a nutshell, the convex hull of a dataset is the smallest convex set that includes all datapoints. Starting from a set of training data, the first approach repeatedly forms the convex hull of transformed data in the latent space upon iterative expansion of the training data fed to the dimensionality reduction algorithm. Upon convergence, the envelope of the final convex geometry can suggest a bound of the so-called feasible responses by a specific class of structures. Figure S12a displays convex hulls for A-GST and C-GST cases with blue and red, respectively, in the 3D latent space. Despite its effectiveness in visually representing binary classified data, there is a major shortcoming with the first approach. The algorithm forcefully expands the convex hull to reach a convexity irrespective of the possible nonconvexity of that class. As a negative result, some datapoints from each class (so-called unfeasible) in the latent space are included in the convex hull of the other class, which degrades the effectiveness of the convex hull approach in distinguishing totally feasible responses. In addition, the convex hull approach does not provide any intuition about the level of feasibility. In the second approach, a quantitative metric is offered by one-class SVM to measure the separability of two classes (i.e., feasible and unfeasible). Basically, a one-class SVM represents a series of separating hyperplanes (corresponding to different levels of unfeasibility) that encompass all instances of a class in the latent space without any convexity restriction. The nonconvex decision boundaries

constructed through the one-class SVM approach approach help us determine how much an object from a different class (e.g., the response of a C-GST metasurface) is close to the one class of interest (e.g., the response of a A-GST metasurface) using an outlier threshold. The graph in Fig. S12b displays several contours (with different colors) surrounding the reddish geometry enclosing A-GST data shown as white dots. Those points located in color-coded counters with negative scores are predicted to be the 10% outliers for the class of A-GST. It is clear from Fig. S12b that majority of observations laying outside the high-confidence region, characterized by positive scores, are C-GST samples (black dots), which are predicted as unfeasible responses for A-GST metasurfaces.

Note 8: Working principle of the wrapper method

The wrapper method as a feature selection algorithm aims to select and rank the most significant and relevant features in a given dataset[48,49]. Beyond that, this technique helps faster training of machine learning algorithms while improves the accuracy of the model and reducing the overfitting problem. The wrapper method follows a greedy search approach by assessing all possible combinations of features against an evaluation criterion. By sequentially adding features staring from an empty feature set, the wrapper algorithm performs cross-validation by calling the evaluation criterion function for each candidate feature subset. In each sequence, the data is first divided into two subsets of training (that contains the selected subset of features and corresponding responses) and testing (that contains the complementary subset of features and corresponding responses). By training a feedforward neural network model with the former subset, the evaluation function returns some measure of distance (or loss) of those predicted by the latter subset. Then, the returned losses are summed up and divided by the number of the test observations leading to the criterion mean, which is considered as a metric for evaluation of the rank of each feature subset. The process continues until adding more feature subsets does not decrease the mean. Here, our

goal is to score each structural parameter given that the evaluation criterion satisfies maximum modulation depth at 1550 nm (with 10% bandwidth) upon switching of the state of GST from amorphous to crystalline. The selected features in order and the corresponding criterion values computed at the consecutive steps are t_{GST}, d_{Au}, p, t_{Al2O3} and [9.61×10^{-5}, 7.30×10^{-5}, 6.03×10^{-5}, 5.52×10^{-5}], respectively, as shown in Fig. S13.

Note 9: Dominant structural parameters revealed by the machine learning approach

As discussed in the main text, feature selection algorithm can define the most influential design parameters without apriori knowledge. To corroborate the potential of this shear mathematical approach, we study the effect of different design parameters on the evolution of fundamental modes of the phase-change metasurface through full-wave electromagnetic simulations. As unveiled in Fig. S14a, variation of t_{GST} significantly affects the spectral evolution of both SR-SPP and LR-SPP modes, where the anti-crossing behavior can occur due to pronounced coupling of these two modes. That is to say the enhancement of the induced magnetic dipole underneath the nanodisk and the coupling strength of the incident light to the in-plane wave are closely linked to this design parameter. In addition, comparison of Figs. S6a and S6b infers the more sensitivity of the optical performance of the metasurface to the characteristics of SR-SPP, which is mainly influenced by changes in d_{Au}, than those of the LR-SPP, whose evolution is basically governed by changes in p. Figure S14b reveals that variation of $t_{\mathrm{Al_2O_3}}$ negligibly affects the overall optical performance of the metasurface validating the results from our feature-selection approach.

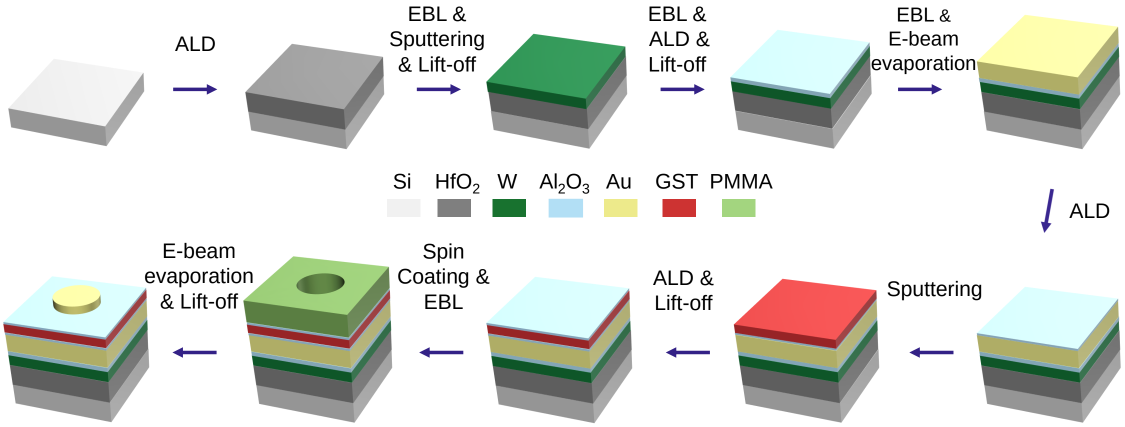

Figure S1: **Fabrication flow diagram of the heterostructure metadevice.** Schematic rendering of the standard process steps for the fabrication of the stacked microheater (excluding large Au pads) and the hybrid plasmonic-PCM metasurface. Detailed explanation of each step is included in Methods.

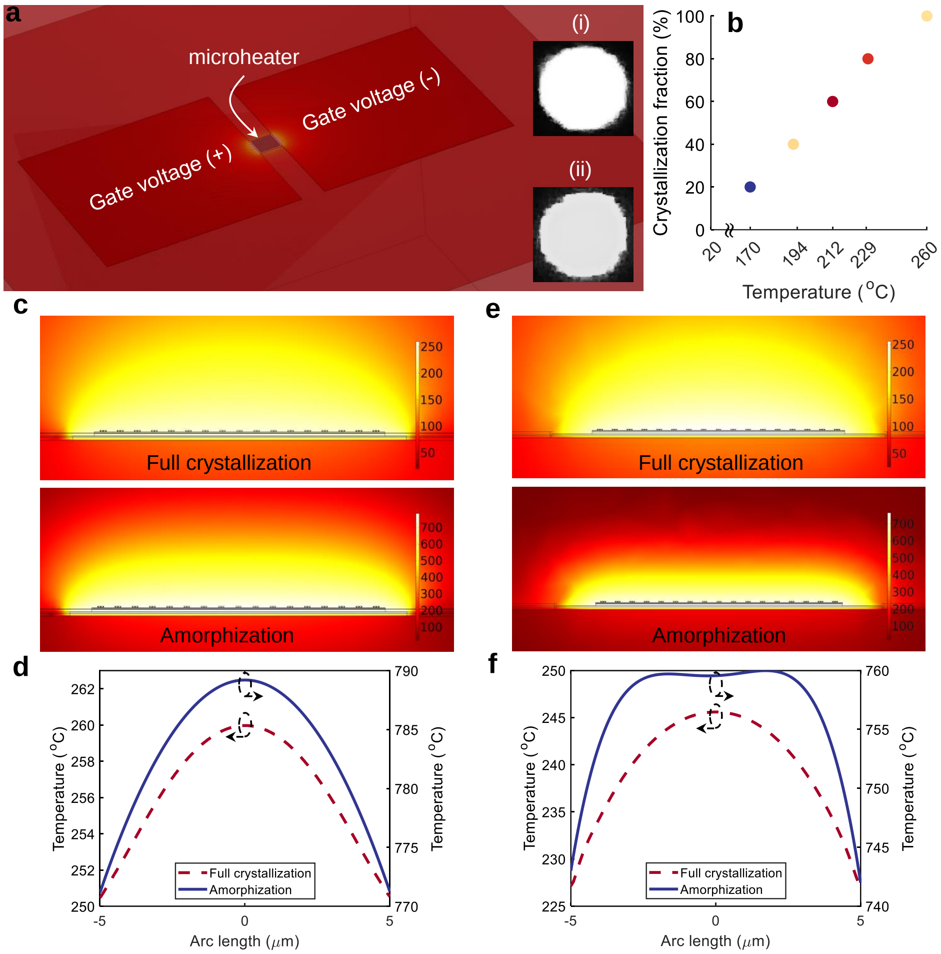

Figure S2: **Simulated electrothermal model of the meta-switch.** (**a**) Schematic of the 3D simulated region in COMSOL Multiphysics. Inset: magnified spatial distribution of the reflected beam from the metasurface shown in Fig. 2a with (i) C-GST and (ii) A-GST at $\lambda = 1440$ nm. Thanks to the fairly even heat profile generated across the microheater at the end of the set/reset pulse, a rather uniform spatial distribution is observable for both cases. (**b**) Crystallization fraction of the GST film with different intermediate states as a function of the induced temperature. The color-coded points are adopted from Fig. 3b. (**c**) The temperature map in the cross section perpendicular to the current flow of the heterostructure meta-switch (see Fig. 1a) and (**d**) the temperature plot on a line in the middle plane of the GST film (across the microheater) at the peak voltage of the set (for full crystallization) and reset (for amorphization) pulse, respectively. (**e**) The temperature map in the cross section parallel to the current flow of the modified heterostructure meta-switch (with an elongated microheater) and (**f**) the temperature plot on a line in the middle plane of the GST film (along the microheater) at the peak voltage of the set (for full crystallization) and reset (for amorphization) pulse, respectively.

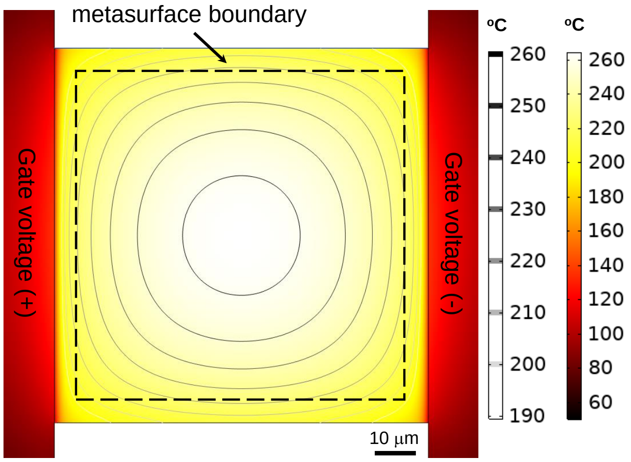

Figure S3: **Simulated electrothermal model for a large-scale microheater with 92 μm × 92 μm size.** The temperature map in a plane at the center of the GST film is shown. The isothermal contours reveal a ∼ 50 °C temperature variation across an 80 μm × 80 μm metasurface at the end of a 200 μs-long 6.7 V set pulse.

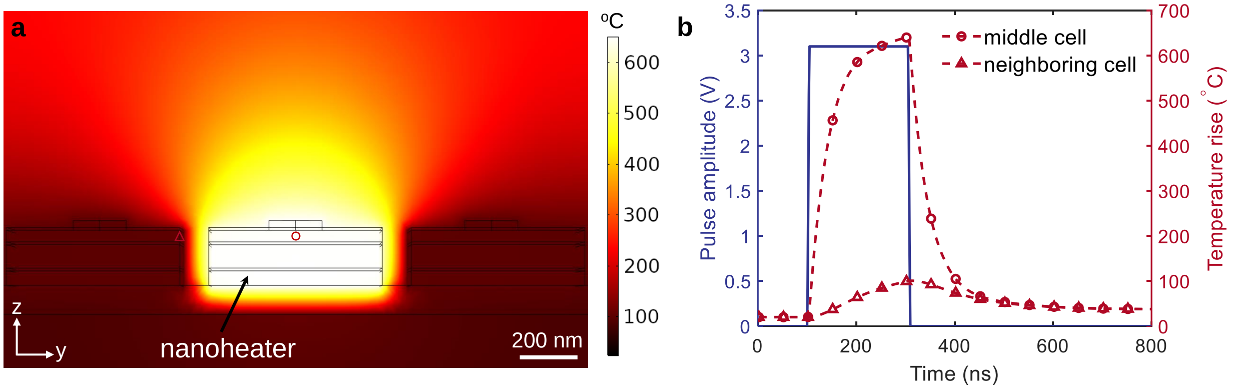

Figure S4: **Simulated electrothermal model for a 1D array of heterostructure ribbons comprising tungsten nanoheaters and the reflective meta-atoms.** (**a**) Simulated temperature distribution at the cross section of the adjacent meta-atoms perpendicular the current flow at the end of the reset pulse (i.e., 300 ns). (**b**) Transient temperature profiles in the center of the middle GST cell as well as the corner of the neighboring cells upon applying the reset pulse. While the middle cell is successfully melted (with temperature above 630 °C) followed by a quenching process (satisfying the high cooling rate of $>$ 1 °C/ns), the maximum temperature experienced by the neighboring cells is well below the crystallization temperature (i.e., ∼ 160 °C).

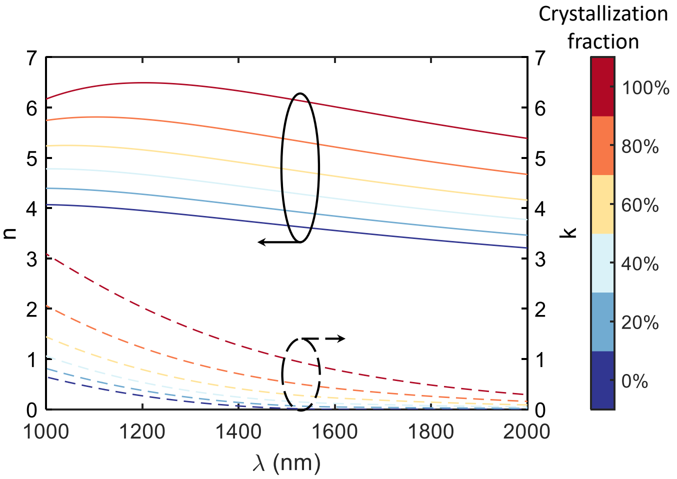

Figure S5: **Optical constants for A-GST, C-GST, and 4 intermediate states of GST.** Refractive index (n, color-coded solid lines) and absorption coefficient (k, color-coded dashed lines) for different crystallization fractions of GST. 0% and 100% correspond to A-GST and C-GST, respectively, while the intermediate states with increasing crystallinity are represented by the crystallization fractions of 20%, 40%, 60%, and 80%. The optical properties of the intermediate states are calculated based on the effective medium theory using ellipsometrically measured A-GST and C-GST data (see Note 2 for details).

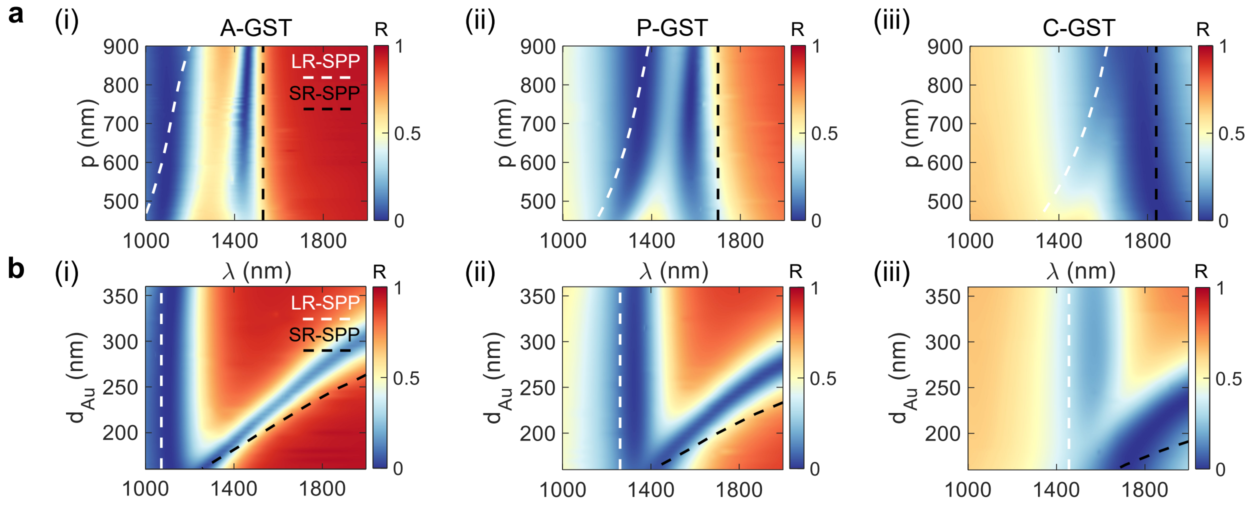

Figure S6: **Spectral evolution of the fundamental modes of the phase-change metasurface.** (**a,b**) Simulated 2D reflectance maps for the metasurface as functions of (**a**) p and (**b**) d_{Au} with (i) A-GST, (ii) P-GST, and (iii) C-GST. The coupling of the incident light to the LR-SPP mode is displayed by the white dashed line while the black dashed line represents the evolution of the SR-SPP mode. $t_{\mathrm{GST}} = 40$ nm, $t_{\mathrm{Al_2O_3}} = 10$ nm, and $t_{\mathrm{Au}} = 35$ nm are fixed while $d_{\mathrm{Au}} = 200$ nm and $p = 600$ nm are chosen in (**a**) and (**b**), respectively.

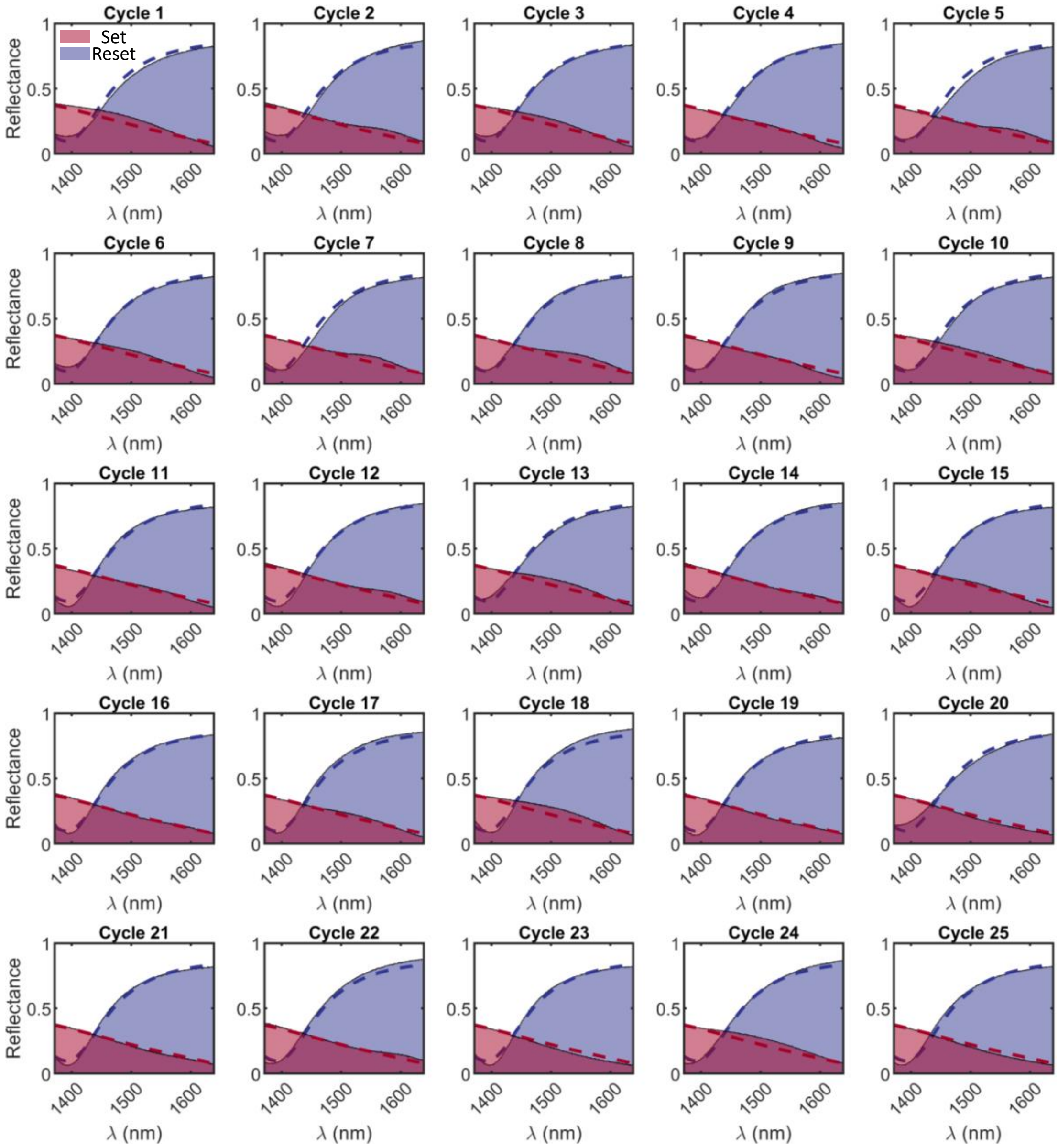

Cycle 1
Cycle 2
Cycle 3
Cycle 4
Cycle 5
Cycle 6
Cycle 7
Cycle 8
Cycle 9
Cycle 10
Cycle 11
Cycle 12
Cycle 13
Cycle 14
Cycle 15
Cycle 16
Cycle 17
Cycle 18
Cycle 19
Cycle 20
Cycle 21
Cycle 22
Cycle 23
Cycle 24
Cycle 25
Set
Reset
Reflectance
λ (nm)
0
0.5
1
1400
1500
1600

Cont'd.

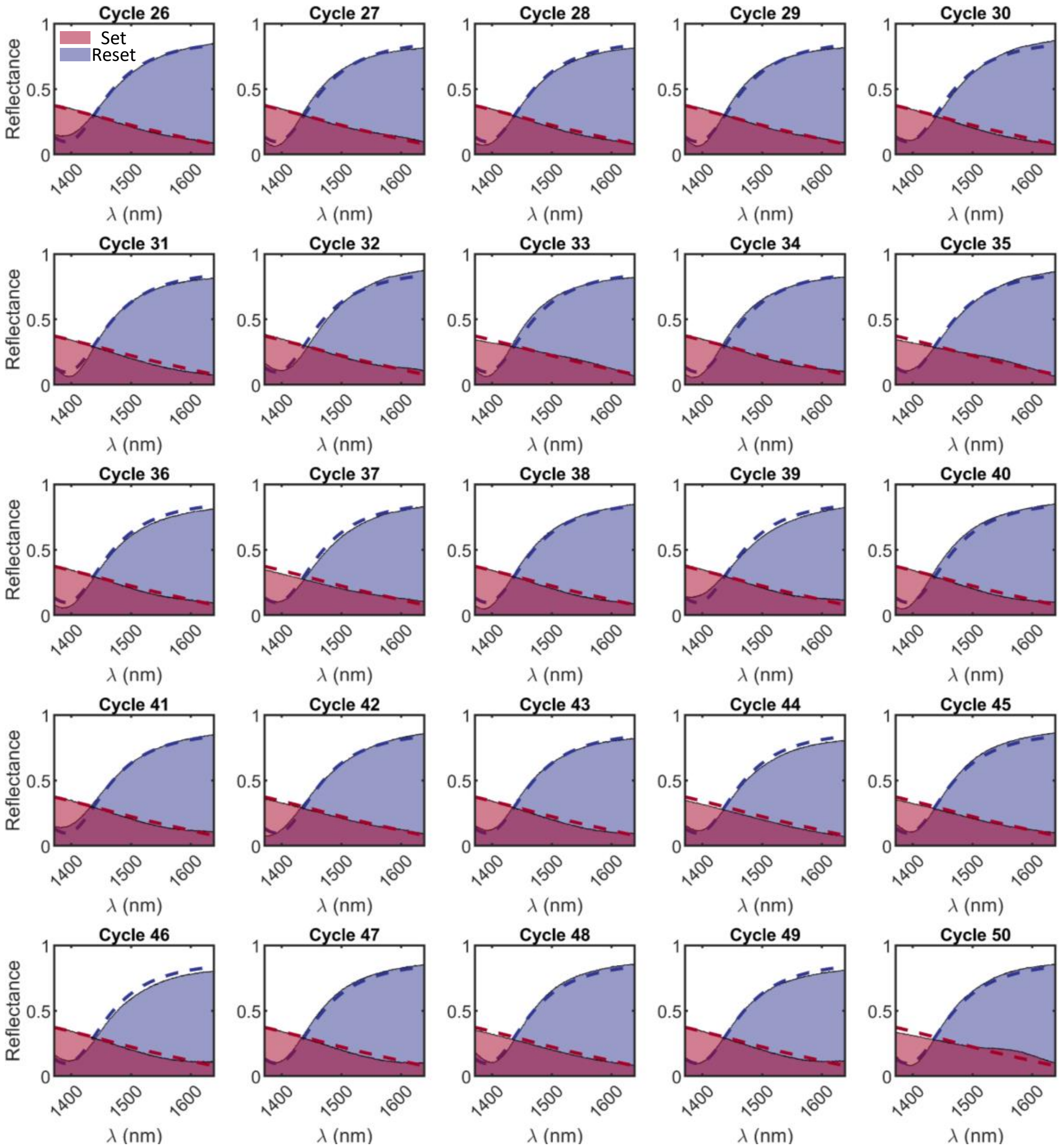

Figure S7: **Repeatability of the phase-change meta-switch.** Reflectance spectra from the studied meta-switch collected after applying individual set/reset pulse for 50 consecutive cycles. The shaded red and blue areas clearly verify the good consistency after each cycle of operation. The average reflectance spectra for A-GST and C-GST (shown in Fig. 2c) are represented by blue and red dashed lines, respectively, as a baseline for a better comparison.

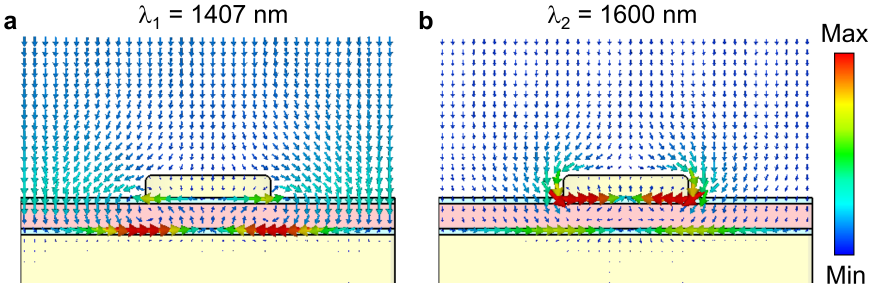

Figure S8: **Full-wave simulation results at the resonance wavelengths of metasurface with 80% crystallization fraction (see Fig. 3a(ii)).** Overlaid flowlines of the Poynting vector exhibit enhancement of the electromagnetic field near the nanodisk in the (**a**) and funneling of incident light into the lossy GST film in (**b**). The structural parameters of the studied metasurface are $p = 600$ nm, $d_{\mathrm{Au}} = 190$ nm, $t_{\mathrm{Au}} = 35$ nm, $t_{\mathrm{GST}} = 40$ nm, and $t_{\mathrm{Al_2O_3}} = 10$ nm with respect to Fig. 1a(ii).

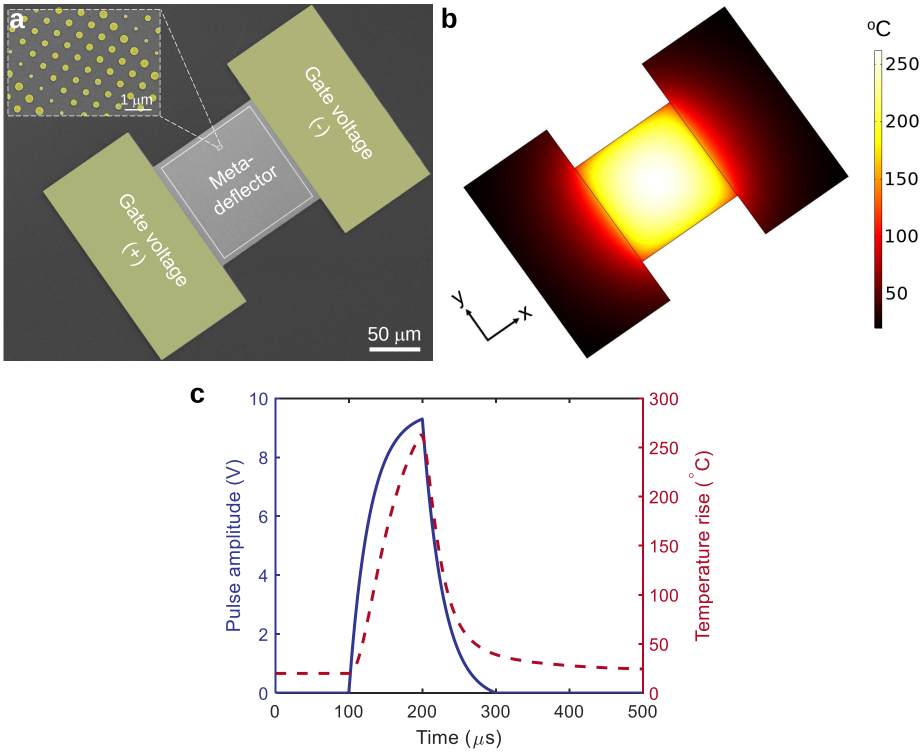

Figure S9: **Meta-deflector configuration and the temperature distribution of the platform.** (**a**) Top view SEM image of an electrically tunable metadevice consisting a phase-change gradient metasurface with an aperture size of 100 μm×100 μm. (**b**) Simulated temperature distribution at a cross section in the center of the GST film of the meta-deflector. (**c**) Real-time voltage of the applied set pulse (solid blue line) and the corresponding temperature response (dashed red lines) in the center of the GST film for full crystallization of the whole film.

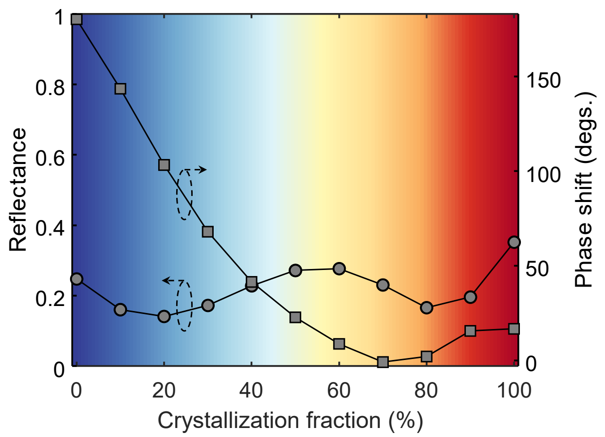

Figure S10: **Phase modulation using programmable phase-change metasurfaces.** Amplitude and phase variations of the reflected light as functions of crystallization fraction at λ =1460 nm. The structural parameters of the studied metasurface are p = 600 nm, d_{Au} = 200 nm, t_{Au} = 35 nm, t_{GST} = 40 nm, and $t_{\mathrm{Al_2O_3}}$ = 10 nm. Upon continuous transition of the GST state, phase shifts > 180° with reflectance variation as low as 0.1 are obtained.

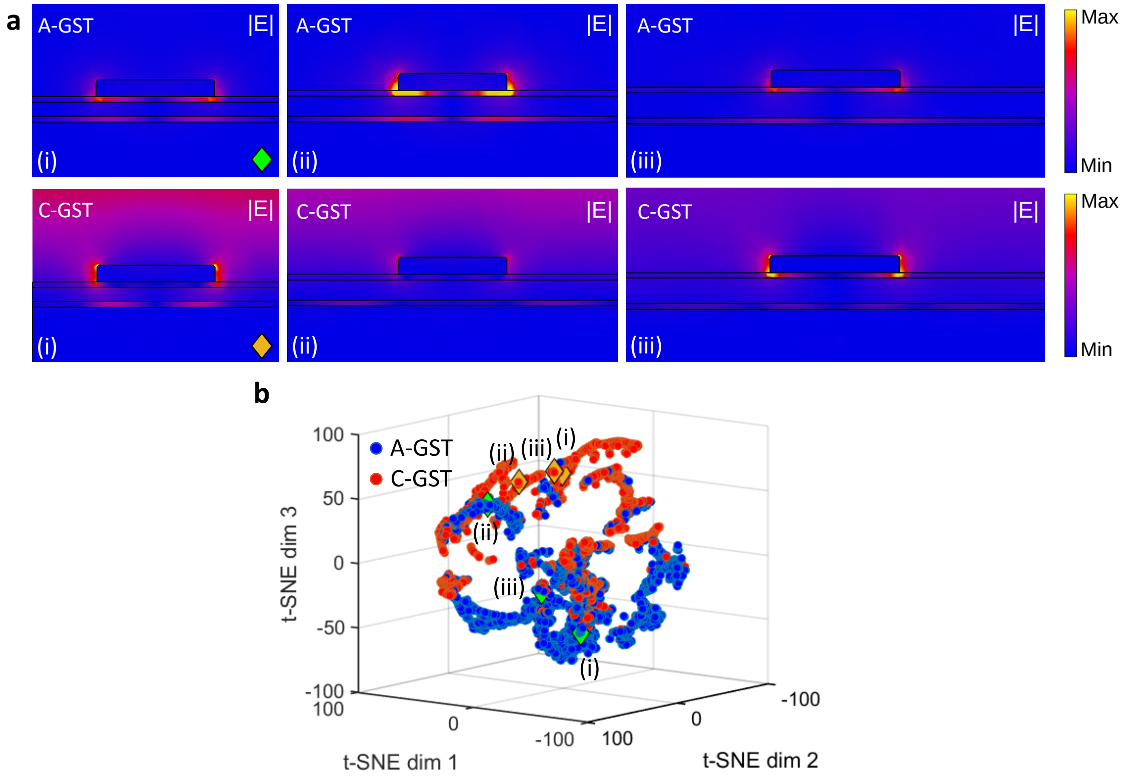

Figure S11: **Latent space representation of the reflection responses of the meta-switch.** (**a**) Inspection of the normalized electric field magnitude at the resonance wavelengths of the meta-switch with A-GST (top row) and C-GST (bottom row) in the $x-z$ plane of a meta-atom. The structural parameters of the studied metasurface are p = 440 nm, d_{Au} = 210 nm, and t_{GST} = 25 nm (first column), p = 580 nm, d_{Au} = 190 nm, t_{GST} = 35 nm (second column), and p = 750 nm, d_{Au} = 230 nm, t_{GST} = 45 nm (third column). (**b**) The embeddings corresponding to A-GST and C-GST in the latent space (rotated with respect to the center of Fig. 5a for a better view). The reduced-dimensional reflectance responses of metasurfaces in panel (**a**) are depicted using color-coded shapes.

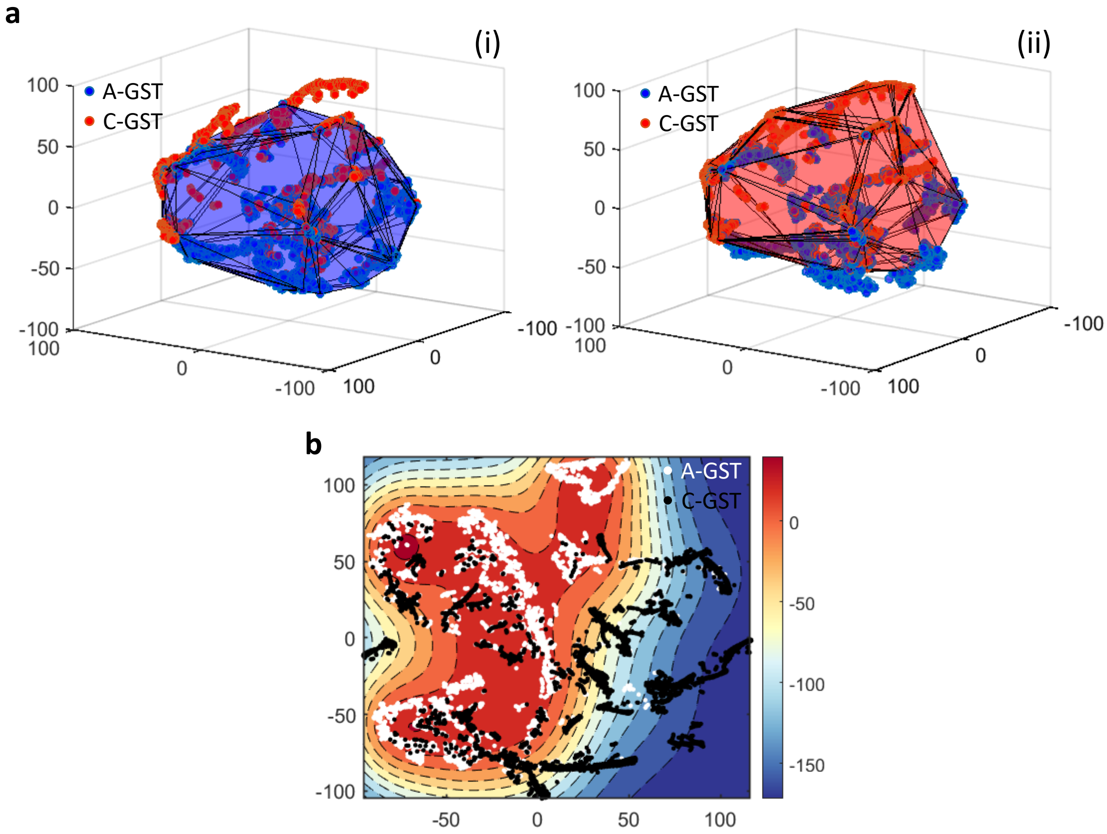

Figure S12: **Representations of the 3D convex hull and one-class SVM for simulated reflectance spectra of the phase-change meta-switch in Fig. 1a.** (**a**) Convex hull of (i) A-GST (with blue facets) and (ii) C-GST (with red facets) metasurfaces representing their feasible responses in the 3D latent space. (**b**) Nonconvex geometry calculated by the one-class SVM algorithm for the A-GST metasurface. The graph shows the separating hyperplanes as a measure of response feasibility in the 2D latent space. The boundary separating 10% observations as outliers from the rest of the data occurs where the contour value is 0. Black points show the responses for the C-GST case. It is clear that for the most part, they fall outside the feasible range of the A-GST metasurface. This point is also observed from panel (a).

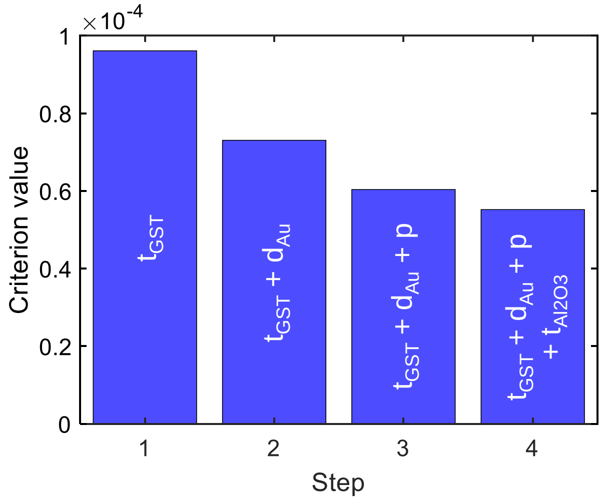

Figure S13: **Criterion values calculated at each step of the wrapper algorithm and the corresponding selected features.**

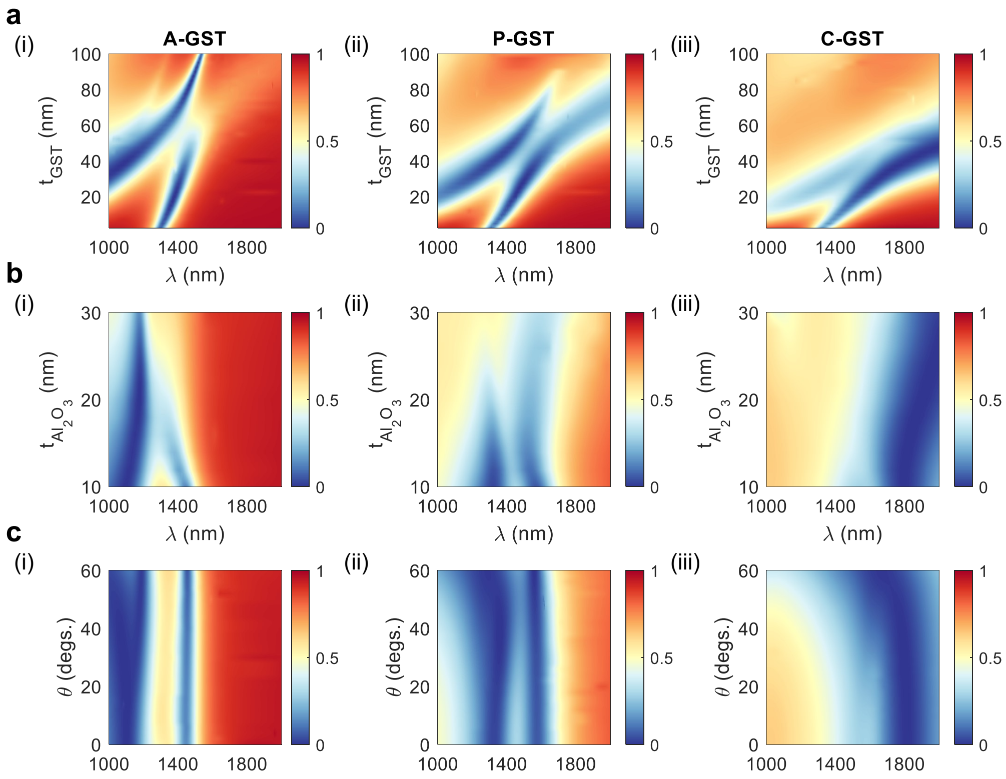

Figure S14: **Structural and angular sensitivity analysis of the phase-change metasurface.** Reflectance colormaps as functions of (**a**) t_{GST}, (**b**) $t_{\mathrm{Al_2O_3}}$, and (**c**) angle of incidence (with respect to the normal direction in Fig. 1a) for the case of (i) A-GST, (ii) P-GST, and (iii) C-GST. The structural parameters are (**a**) $t_{\mathrm{Al_2O_3}} = 10$ nm, (**b**) $t_{\mathrm{GST}} = 40$ nm, and (**c**) $t_{\mathrm{GST}} = 40$ nm, and $t_{\mathrm{Al_2O_3}} = 10$ nm with respect to Fig. 1a(ii). $p = 600$ nm, $d_{\mathrm{Au}} = 200$ nm, and $t_{\mathrm{Au}} = 35$ nm are fixed in all simulations.

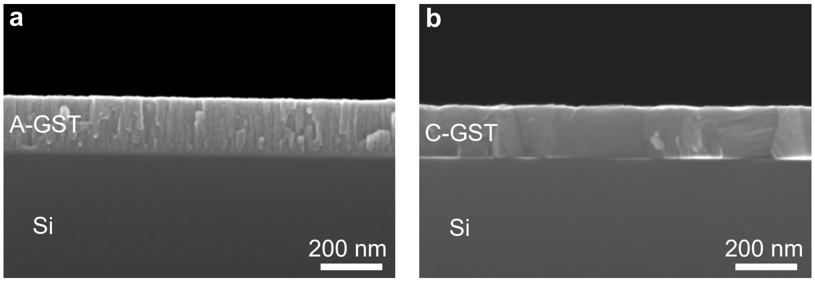

Figure S15: **Morphological analysis of blanket layers of A-GST and C-GST.** Cross section SEM images of a 200-nm-thick (**a**) A-GST and (**b**) C-GST films. The physical thickness of the GST layer uniformly shrinks by $\sim 5\%$ due to an increase in the film density by the crystallization process, which is in agreement with the existing experimental results [50].

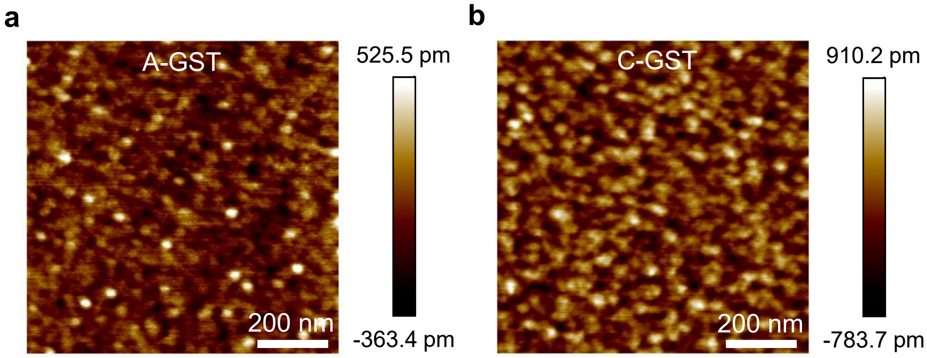

Figure S16: **Surface characteristics of A-GST and C-GST films.** Atomic force microscope images from the surface of a film of (**a**) A-GST and (**b**) C-GST. The surface of the GST film experiences slight roughness after the crystallization process.

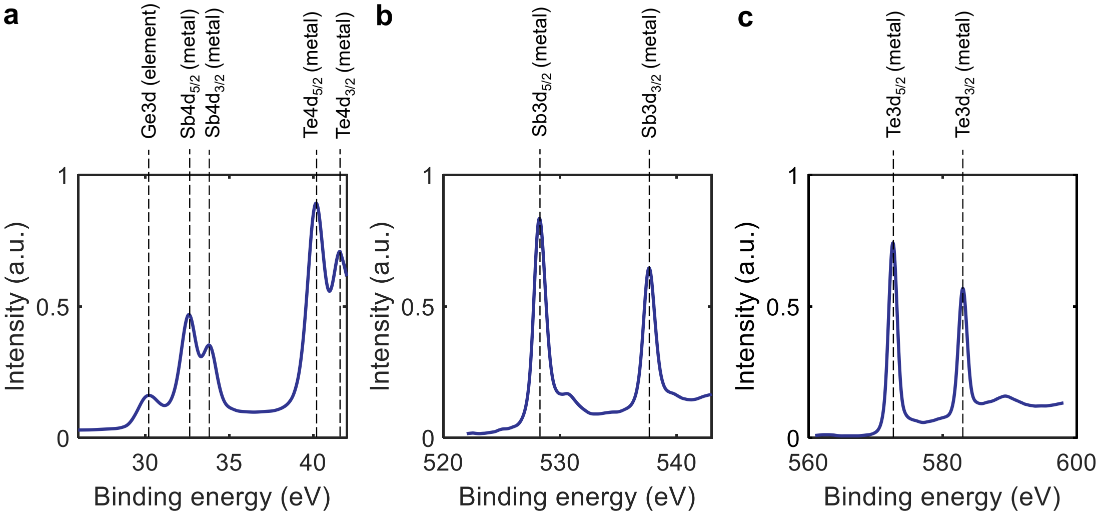

Figure S17: **X-ray photoelectron spectroscopy of the GST film.** XPS spectra of (**a**) Ge 3d, Sb 4d and Te 4d, (**b**) Sb 3d, and (**c**) Te 3d obtained from an A-GST film.

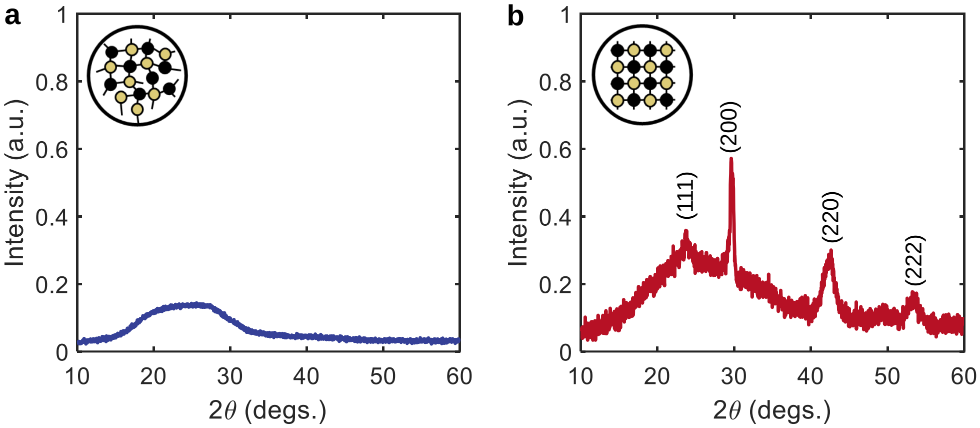

Figure S18: **X-ray diffraction spectra of the GST film.** Each diffraction peak of C-GST is attributed to the scattering from a specific set of parallel planes of atoms. Inset: a generic scheme of the atomic distribution of (**a**) A-GST and (**b**) C-GST.

References

(1) Hugon, M.; Varniere, F.; Agius, B.; Froment, M.; Arena, C.; Bessot, J. Stresses, microstructure and resistivity of thin tungsten films deposited by RF magnetron sputtering. *Applied surface science* **1989**, *38*, 269–285.

(2) Vink, T.; Walrave, W.; Daams, J.; Dirks, A.; Somers, M.; Van den Aker, K. Stress, strain, and microstructure in thin tungsten films deposited by dc magnetron sputtering. *Journal of applied physics* **1993**, *74*, 988–995.

(3) Yeoh, P.; Ma, Y.; Cullen, D. A.; Bain, J. A.; Skowronski, M. Thermal-gradient-driven elemental segregation in Ge2Sb2Te5 phase change memory cells. *Applied Physics Letters* **2019**, *114*, 163507.

(4) Krusin-Elbaum, L.; Ahn, K.; Souk, J.; Ting, C.; Nesbit, L. Effects of deposition methods on the temperature-dependent resistivity of tungsten films. *Journal of Vacuum Science & Technology A: Vacuum, Surfaces, and Films* **1986**, *4*, 3106–3110.

(5) Chen, X.; Chen, Y.; Yan, M.; Qiu, M. Nanosecond photothermal effects in plasmonic nanostructures. *ACS nano* **2012**, *6*, 2550–2557.

(6) Li, X.; Park, W.; Wang, Y.; Chen, Y. P.; Ruan, X. Reducing interfacial thermal resistance between metal and dielectric materials by a metal interlayer. *Journal of Applied Physics* **2019**, *125*, 045302.

(7) Fallica, R.; Battaglia, J.-L.; Cocco, S.; Monguzzi, C.; Teren, A.; Wiemer, C.; Varesi, E.; Cecchini, R.; Gotti, A.; Fanciulli, M. Thermal and electrical characterization of materials for phase-change memory cells. *Journal of Chemical & Engineering Data* **2009**, *54*, 1698–1701.

(8) Reifenberg, J. P.; Panzer, M. A.; Kim, S.; Gibby, A. M.; Zhang, Y.; Wong, S.; Wong, H.-

S. P.; Pop, E.; Goodson, K. E. Thickness and stoichiometry dependence of the thermal conductivity of GeSbTe films. *Applied Physics Letters* **2007**, *91*, 111904.

(9) Reifenberg, J.; Pop, E.; Gibby, A.; Wong, S.; Goodson, K. Multiphysics modeling and impact of thermal boundary resistance in phase change memory devices. Thermal and Thermomechanical Proceedings 10th Intersociety Conference on Phenomena in Electronics Systems, 2006. ITHERM 2006. 2006; pp 106–113.

(10) Lee, J.; Kim, S.; Jeyasingh, R.; Asheghi, M.; Wong, H.-S. P.; Goodson, K. E. Microthermal stage for electrothermal characterization of phase-change memory. *IEEE electron device letters* **2011**, *32*, 952–954.

(11) Olson, D. H.; Freedy, K. M.; McDonnell, S. J.; Hopkins, P. E. The influence of titanium adhesion layer oxygen stoichiometry on thermal boundary conductance at gold contacts. *Applied Physics Letters* **2018**, *112*, 171602.

(12) Scott, E. A.; Gaskins, J. T.; King, S. W.; Hopkins, P. E. Thermal conductivity and thermal boundary resistance of atomic layer deposited high-k dielectric aluminum oxide, hafnium oxide, and titanium oxide thin films on silicon. *APL Materials* **2018**, *6*, 058302.

(13) Xu, M. Study of the crystallization dynamics and threshold voltage of phase change materials for use in reconfigurable RF switches and non-volatile memories. Ph.D. thesis, Carnegie Mellon University, 2017.

(14) Yamada, N.; Ohno, E.; Nishiuchi, K.; Akahira, N.; Takao, M. Rapid-phase transitions of GeTe-Sb2Te3 pseudobinary amorphous thin films for an optical disk memory. *Journal of Applied Physics* **1991**, *69*, 2849–2856.

(15) Pop, E. Energy dissipation and transport in nanoscale devices. *Nano Research* **2010**, *3*, 147–169.

(16) Cahill, D. G.; Ford, W. K.; Goodson, K. E.; Mahan, G. D.; Majumdar, A.; Maris, H. J.; Merlin, R.; Phillpot, S. R. Nanoscale thermal transport. *Journal of applied physics* **2003**, *93*, 793–818.

(17) Xiong, F.; Liao, A. D.; Estrada, D.; Pop, E. Low-power switching of phase-change materials with carbon nanotube electrodes. *Science* **2011**, *332*, 568–570.

(18) Shirmanesh, G. K.; Sokhoyan, R.; Wu, P. C.; Atwater, H. A. Electro-optically tunable multifunctional metasurfaces. *ACS nano* **2020**, *14*, 6912–6920.

(19) Aspnes, D. Local-field effects and effective-medium theory: a microscopic perspective. *American Journal of Physics* **1982**, *50*, 704–709.

(20) Shportko, K.; Kremers, S.; Woda, M.; Lencer, D.; Robertson, J.; Wuttig, M. Resonant bonding in crystalline phase-change materials. *Nature materials* **2008**, *7*, 653–658.

(21) Raoux, S.; Xiong, F.; Wuttig, M.; Pop, E. Phase change materials and phase change memory. *MRS bulletin* **2014**, *39*, 703–710.

(22) Wong, H.-S. P.; Raoux, S.; Kim, S.; Liang, J.; Reifenberg, J. P.; Rajendran, B.; Asheghi, M.; Goodson, K. E. Phase change memory. *Proceedings of the IEEE* **2010**, *98*, 2201–2227.

(23) Lee, B.-S.; Abelson, J. R.; Bishop, S. G.; Kang, D.-H.; Cheong, B.-k.; Kim, K.-B. Investigation of the optical and electronic properties of Ge 2 Sb 2 Te 5 phase change material in its amorphous, cubic, and hexagonal phases. *Journal of Applied Physics* **2005**, *97*, 093509.

(24) Raoux, S. Phase change materials. *Annual Review of Materials Research* **2009**, *39*, 25–48.

(25) Rios, C.; Stegmaier, M.; Cheng, Z.; Youngblood, N.; Wright, C. D.; Pernice, W. H.;

Bhaskaran, H. Controlled switching of phase-change materials by evanescent-field coupling in integrated photonics. *Optical Materials Express* **2018**, *8*, 2455–2470.

(26) Loke, D. K.; Skelton, J. M.; Lee, T. H.; Zhao, R.; Chong, T.-C.; Elliott, S. R. Ultrafast nanoscale phase-change memory enabled by single-pulse conditioning. *ACS applied materials & interfaces* **2018**, *10*, 41855–41860.

(27) Simpson, R. E.; Fons, P.; Kolobov, A. V.; Fukaya, T.; Krbal, M.; Yagi, T.; Tominaga, J. Interfacial phase-change memory. *Nature nanotechnology* **2011**, *6*, 501–505.

(28) Wang, R.; Calarco, R.; Arciprete, F.; Bragaglia, V. Epitaxial growth of GeTe/Sb2Te3 superlattices. *Materials Science in Semiconductor Processing* **2022**, *137*, 106244.

(29) Pore, V.; Hatanpaa, T.; Ritala, M.; Leskela, M. Atomic layer deposition of metal tellurides and selenides using alkylsilyl compounds of tellurium and selenium. *Journal of the American Chemical Society* **2009**, *131*, 3478–3480.

(30) Nam, S.-W.; Chung, H.-S.; Lo, Y. C.; Qi, L.; Li, J.; Lu, Y.; Johnson, A. C.; Jung, Y.; Nukala, P.; Agarwal, R. Electrical wind force–driven and dislocation-templated amorphization in phase-change nanowires. *Science* **2012**, *336*, 1561–1566.

(31) Sarwat, S. G. Materials science and engineering of phase change random access memory. *Materials Science and Technology* **2017**, *33*, 1890–1906.

(32) Zheng, J.; Fang, Z.; Wu, C.; Zhu, S.; Xu, P.; Doylend, J. K.; Deshmukh, S.; Pop, E.; Dunham, S.; Li, M., et al. Nonvolatile electrically reconfigurable integrated photonic switch enabled by a silicon PIN diode heater. *Advanced Materials* **2020**, *32*, 2001218.

(33) Tuma, T.; Pantazi, A.; Le Gallo, M.; Sebastian, A.; Eleftheriou, E. Stochastic phase-change neurons. *Nature nanotechnology* **2016**, *11*, 693.

(34) Li, X.; Youngblood, N.; Ríos, C.; Cheng, Z.; Wright, C. D.; Pernice, W. H.;

Bhaskaran, H. Fast and reliable storage using a 5 bit, nonvolatile photonic memory cell. *Optica* **2019**, *6*, 1–6.

(35) Ríos, C.; Stegmaier, M.; Hosseini, P.; Wang, D.; Scherer, T.; Wright, C. D.; Bhaskaran, H.; Pernice, W. H. Integrated all-photonic non-volatile multi-level memory. *Nature Photonics* **2015**, *9*, 725.

(36) Papandreou, N.; Pozidis, H.; Pantazi, A.; Sebastian, A.; Breitwisch, M.; Lam, C.; Eleftheriou, E. Programming algorithms for multilevel phase-change memory. 2011 IEEE International Symposium of Circuits and Systems (ISCAS). 2011; pp 329–332.

(37) Huang, Y.-W.; Lee, H. W. H.; Sokhoyan, R.; Pala, R. A.; Thyagarajan, K.; Han, S.; Tsai, D. P.; Atwater, H. A. Gate-tunable conducting oxide metasurfaces. *Nano letters* **2016**, *16*, 5319–5325.

(38) Van der Maaten, L.; Hinton, G. Visualizing data using t-SNE. *Journal of machine learning research* **2008**, *9*.

(39) Van Der Maaten, L. Accelerating t-SNE using tree-based algorithms. *The Journal of Machine Learning Research* **2014**, *15*, 3221–3245.

(40) Gisbrecht, A.; Hammer, B. Data visualization by nonlinear dimensionality reduction. *Wiley Interdisciplinary Reviews: Data Mining and Knowledge Discovery* **2015**, *5*, 51–73.

(41) Bunte, K.; Biehl, M.; Hammer, B. A general framework for dimensionality-reducing data visualization mapping. *Neural Computation* **2012**, *24*, 771–804.

(42) Wattenberg, M.; Viégas, F.; Johnson, I. How to use t-SNE effectively. *Distill* **2016**, *1*, e2.

(43) Becht, E.; McInnes, L.; Healy, J.; Dutertre, C.-A.; Kwok, I. W.; Ng, L. G.; Ginhoux, F.;

Newell, E. W. Dimensionality reduction for visualizing single-cell data using UMAP. *Nature biotechnology* **2019**, *37*, 38–44.

(44) Linderman, G. C.; Rachh, M.; Hoskins, J. G.; Steinerberger, S.; Kluger, Y. Fast interpolation-based t-SNE for improved visualization of single-cell RNA-seq data. *Nature methods* **2019**, *16*, 243–245.

(45) Kobak, D.; Berens, P. The art of using t-SNE for single-cell transcriptomics. *Nature communications* **2019**, *10*, 1–14.

(46) Linderman, G. C.; Steinerberger, S. Clustering with t-SNE, provably. *SIAM Journal on Mathematics of Data Science* **2019**, *1*, 313–332.

(47) Kiarashinejad, Y.; Zandehshahvar, M.; Abdollahramezani, S.; Hemmatyar, O.; Pourabolghasem, R.; Adibi, A. Knowledge discovery in nanophotonics using geometric deep learning. *Advanced Intelligent Systems* **2020**, *2*, 1900132.

(48) Kohavi, R.; John, G. H. Wrappers for feature subset selection. *Artificial intelligence* **1997**, *97*, 273–324.

(49) Guyon, I.; Elisseeff, A. An introduction to variable and feature selection. *Journal of machine learning research* **2003**, *3*, 1157–1182.

(50) Li, P.; Yang, X.; Maß, T. W.; Hanss, J.; Lewin, M.; Michel, A.-K. U.; Wuttig, M.; Taubner, T. Reversible optical switching of highly confined phonon–polaritons with an ultrathin phase-change material. *Nature materials* **2016**, *15*, 870–875.
